# Haplotype Variation of Flowering Time Genes of Sugar Beet and Its Wild Relatives and the Impact on Life Cycle Regimes

**DOI:** 10.3389/fpls.2017.02211

**Published:** 2018-01-04

**Authors:** Nadine Höft, Nadine Dally, Mario Hasler, Christian Jung

**Affiliations:** ^1^Plant Breeding Institute, Christian-Albrechts-University of Kiel, Kiel, Germany; ^2^Lehrfach Variationsstatistik, Christian-Albrechts-University of Kiel, Kiel, Germany

**Keywords:** *Beta vulgaris*, ssp. *maritima*, vernalization, bolting, phenological development

## Abstract

The species *Beta vulgaris* encompasses wild and cultivated members with a broad range of phenological development. The annual life cycle is commonly found in sea beets (ssp. *maritima*) from Mediterranean environments which germinate, bolt, and flower within one season under long day conditions. Biennials such as the cultivated sugar beet (*B. vulgaris* ssp. *vulgaris*) as well as sea beets from northern latitudes require prolonged exposure to cold temperature over winter to acquire floral competence. Sugar beet is mainly cultivated for sugar production in Europe and is likely to have originated from sea beet. Flowering time strongly affects seed yield and yield potential and is thus a trait of high agronomic relevance. Besides environmental cues, there are complex genetic networks known to impact life cycle switch in flowering plants. In sugar beet, *BTC1, BvBBX19, BvFT1*, and *BvFT2* are major flowering time regulators. In this study, we phenotyped plants from a diversity *Beta* panel encompassing cultivated and wild species from different geographical origin. Plants were grown under different day length regimes with and without vernalization. Haplotype analysis of *BTC1, BvBBX19, BvFT1*, and *BvFT2* was performed to identify natural diversity of these genes and their impact on flowering. We found that accessions from northern latitudes flowered significantly later than those from southern latitudes. Some plants did not flower at all, indicating a strong impact of latitude of origin on life cycle. Haplotype analysis revealed a high conservation of the CCT-, REC-, BBX-, and PEBP-domains with regard to SNP occurrence. We identified sequence variation which may impact life cycle adaptation in beet. Our data endorse the importance of *BTC1* in the domestication process of cultivated beets and contribute to the understanding of distribution and adaption of *Beta* species to different life cycle regimes in response to different environments. Moreover, our data provide a resource for haplotypes identified for the major floral regulators in beet.

## Introduction

To ensure reproductive and therewith evolutionary success, flowering plants have developed different life cycles. Sea beets (*Beta vulgaris* L. ssp. *maritima*) are wild relatives of sugar beet (*B. vulgaris* L. ssp. *vulgaris*). Annual sea beets from Mediterranean environments germinate, bolt, and flower within one season under long days, whereas most sea beets from northern latitudes are biennial. They need prolonged exposure to cold temperatures (typically during winter) to acquire a floral competent state. Besides, there are perennial sea beets mostly from Northern Europe which exhibit an iteroparous life cycle (Hautekèete et al., 2002). While iteroparous beets revert to vegetative growth after reproduction, annual and biennial beets are semelparous and die after reproduction (Hautekèete et al., 2001). The onset of floral transition in beets is indicated by the elongation of the main shoot which is commonly referred to as “bolting.” In sugar beet cultivation, early bolting (without vernalization) is a trait of high agronomic relevance because it causes severe yield loss. The genetic control of photoperiodic flowering has been elucidated in the model plant *Arabidopsis thaliana* and many of the identified genes are structurally conserved in all known plants (Capovilla et al., 2014; Pajoro et al., 2014; Blümel et al., 2015).

In beet, major components of the photoperiodic pathway have been identified. The bolting locus *BOLTING TIME CONTROL 1* (*BTC1*) determines the annual life (Pin et al., 2012). *BTC1* was identified as a pseudo-response regulator (PRR) gene, sharing sequence homology with the *PSEUDO RESPONSE REGULATOR* 7 (*PRR7*) gene from *A. thaliana*. It encodes for a protein carrying a response regulator receiver (REC) and a CONSTANS, CONSTANS-Like, and TOC1 (CCT) domain. Beets carrying the dominant *BTC1* allele mainly reveal an annual growth habit such as most sea beet genotypes, while beets carrying the recessive *btc1* allele exhibit a biennial life cycle (Pin et al., 2012). Two *FLOWERING LOCUS T* (*FT*) genes, *BvFT1*, and *BvFT2* which are homologous to the Arabidopsis *FT*, were discovered acting downstream of *BTC1* (Pin et al., 2010). *BvFT1* and *BvFT2*, both belonging to the phosphatidylethanolamine-binding protein (PEBP) gene family, have evolved antagonistic functions. While *BvFT2* promotes flowering and is required for floral development, *BvFT1* acts as a floral repressor. Pin et al. (2012) proposed a model for life cycle control in beet with *BTC1* acting upstream of *BvFT1* and *BvFT2*. In annual beets, the dominant *BTC1* allele represses *BvFT1* and concurrently activates *BvFT2* to induce bolting and flowering. On the contrary, in biennial beets the expression of the recessive *btc1* allele is increasing gradually to a decreasing expression of *BvFT1* during vernalization, enabling the promotion of *BvFT2* expression to initiate flowering. The recent discovery of another bolting time regulator *BvBBX19* encoding for a DOUBLE B-BOX TYPE ZINC FINGER protein extended the model for bolting time regulation in beet (Dally et al., 2014). *BvBBX19* is diurnally regulated and acts epistatically over *BTC1* upstream of *BvFT1* and *BvFT2*. Interestingly, *BTC1* transcription was reduced in *BvBBX19* mutants suggesting a physical interaction of both proteins to jointly regulate *BvFT1* and *BvFT2* (Dally et al., 2014). In addition to those major regulators, several *CONSTANS-LIKE* (*COL*) genes have been detected, differing by their zinc-finger (B-Box) and CCT domains (Chia et al., 2008; Dally et al., 2014). To date, only *BvCOL1* has been functionally characterized by overexpression in Arabidopsis (Chia et al., 2008) but it was excluded as a functional ortholog of *CO* due to non-typical expression profile. Hébrard et al. (2013) compared gene expression and DNA methylation profiles of bolting-resistant and bolting-sensitive beet genotypes after vernalization and determined 169 differentially expressed genes and 111 differentially methylated regions as putative bolting loci. The *SBT-9*/*BR1* locus was discovered to control bolting resistance after winter (Pfeiffer et al., 2014), where a homolog of the Arabidopsis *CLEAVAGE AND POLYADENYLATION SPECIFIC FACTOR 73-I* (*CPSF73-I*) was identified as the most promising candidate gene (Tränkner et al., 2016). Recently, Tränkner et al. (2017) proposed that two QTL contribute to variation in seasonal bolting. Besides *SBT-9*/*BR1*, the *SBT-4* locus was elucidated to majorly control seasonal bolting and *BvFT2* was suggested as a candidate gene.

The adaptation to different environments is of central importance for the evolutionary success in flowering plants. In *Beta* species, adaptation to different geographical regions is processed through the evolution of different life cycles (Hautekèete et al., 2002). It was suggested that the domestication of sugar beet involved the selection of a rare partial loss-of-function allele of *BTC1*, which alters the plant's response to long day conditions (Pin et al., 2012). A *BTC1* haplotype analysis of a large number of *Beta* accessions and cultivars revealed eleven haplotypes divided into two classes, “annuals” (*BTC1*_*d*−*k*_) and “biennials” (*btc1*_*a*−*c*_). These two classes mainly differ by six non-synonymous single-nucleotide polymorphisms (SNPs) as well as a large insertion (~28 kb) within the promoter of biennial *btc1* alleles (Pin et al., 2012). Intriguingly, vast majority of cultivated beets carry the recessive *btc1*_*a*_ allele while sea beets mainly exhibited *BTC1* alleles from the “annual” class.

In contrast, information about *BvBBX19, BvFT1*, and *BvFT2* haplotypes and their abundance among wild and cultivated species is lacking so far. This study aims to understand the role of the four major *Beta* flowering time regulators *BTC1, BvFT1, BvFT2*, and *BvBBX19* on the adaptation to different environments. We assumed that sequence variations within the coding region of these genes have a major impact on phenological development. Consequently, a non-random distribution of haplotypes across accessions from different geographical origin was expected. Moreover, we reasoned that life cycle changes follow a latitudinal cline. For this purpose, 29 *Beta* accessions from different geographical origin were grown under standardized conditions and the onset of bolting was recorded. The coding regions of *BTC1, BvFT1, BvFT2*, and *BvBBX19* were sequenced from all accessions and found high variation within *BTC1*, whereas sequence variation among the other genes was low. A relationship between haplotype variation and life cycle regime could be established. Cultivated beets carry similar combinations of their *BTC1, BvBBX19, BvFT1*, and *BvFT2* haplotypes while sea beets displayed a much higher heterogeneity. These results demonstrate that haplotype variations of flowering time regulator genes are main drivers of the adaptive evolution of *Beta* species and the domestication of cultivated beet.

## Materials and methods

### Plant material, growth conditions, and phenotypic analysis

*Beta* accessions were selected based on geographical diversity and expected bolting characteristics (annual and biennial; Table 1). Seeds were sown in 9 cm^2^ pots and plants were grown and phenotyped in a climate chamber with 10 plants per accession under different experimental conditions: 22 h of light, 20°C [experiment 1 (E1)], 16 h of light, 20°C [experiment 2 (E2)], and 22 h of light, 20°C interrupted by a cold treatment at 4°C for 3 months [experiment 3 (E3)]. Plants were watered every second day. In experiment 3 plants were fertilized twice, after 119 days directly before vernalization as well as after 210 days directly after vernalization with PERIMOR. The light intensity was held at 315 μmol m^−2^s^−1^ and the humidity was about 70%. Bolting (BBCH 51) and flowering (BBCH 60) was recorded according to Meier et al., 1993). Without vernalization, 16 weeks after sowing, plants were classified as annual (bolting) or biennial (non-bolting; experiment 1 and 2). Plants which did not bolt 16 weeks after vernalization were classified as “never bolting” (experiment 3).

**Table 1:** Plant material used in this study.

**Variety**	**Species name**	**Seed code**	**Geographical Origin**	**Latitude (0°'N)**
Wild beet	*B. vulgaris* ssp. *maritima*	080287	Ireland	53.0
		080461	Denmark	56.0
		080468	Egypt	27.0
		080437	Pakistan	31.0
		080418	India	21.0
		100539	Germany	51.0
		991971	Greece	39.0
		080260	Netherlands	52.0
		930034	Spain	40.0
		112787	France	46.0
		112823	Great Britain	54.0
		080538	Great Britain	54.0
Sugar beet	*B. vulgaris ssp. vulgaris*	090023	Germany	51.0
		930176	Germany	51.0
		130333	Germany	51.0
		100043	Germany	51.0
		001684	Germany	51.0
		080394	Iran	32.0
		930181	USA	45.0
		080384	Turkey	39.0
		091645	Germany	51.0
Fodder beet	*B. vulgaris ssp. vulgaris*	080281	Germany	51.0
		080313	Greece	39.0
		080396	Iran	32.0
Red table beet	*B. vulgaris ssp. vulgaris*	092312	Russia	60.0
		080339	France	45.5
Leaf beet	*B. vulgaris ssp.vulgaris*	080238	Iraq	33.0
		081845	China	35.0
		092459	Italy	42.0

### Molecular analysis

The coding region of the flowering time genes *BvBBX19, BTC1, BvFT1*, and *BvFT2* was amplified by PCR. Primers and PCR conditions are listed in Supplementary Tables 1, 2. *In silico* prediction of the coding gene structures of *BTC1, BvBBX19, BvFT1*, and *BvFT2* and primer positions are indicated in Supplementary Figure 1. DNA was isolated from leaves using the CTAB method (Rogers and Bendich, 1985) with slight modifications. PCRs were performed for single plants and PCR products of all plants of the same accession were diluted to an equal concentration and pooled. Sanger sequencing of all pools was performed at the Institute of Clinical Molecular Biology (IKMB, CAU Kiel). Sequence analysis was done with the CLC Main Workbench 6.9 (CLC bio, Aarhus, Denmark) and the DNASTAR Lasergene SeqMan Pro (DNASTAR Inc., Madison, USA) program packages. Allelic haplotypes were defined by aligning obtained sequences of the amplified fragments and checking for single nucleotide polymorphisms (SNPs) and insertion/deletion polymorphisms. Pooled sequences were blasted against the beet reference sequence (KWS2320Refseq0.9) (Dohm et al., 2013) using the BLASTN function of the CLC Main Workbench 6.9. All SNP positions were numbered beginning with the translation start site. The evaluation of SNPs and their positions was performed according to the IUPAC code (Johnson, 2010; Supplementary Table 3). Polymorphisms were categorized as synonymous (no impact on the amino acid sequence) or non-synonymous.

### Statistical analysis

The software R (R Development Core Team, 2015) was used for statistical analysis. The data evaluation started with the definition of an appropriate statistical mixed model (Laird and Ware, 1982). The data were assumed to be normally distributed and to be heteroscedastic due to the different levels of environments (experiments) and latitude. These assumptions are based on a graphical residual analysis. The statistical model included a pseudo factor (Schaarschmidt and Vaas, 2009), consisting of the actual factors experiment (E1, E2, E3), latitude (21°N-60°N) and varieties (sea beet, sugar beet, table beet, fodder beet, and leaf beet). This pseudo factor was necessary because the actual factors are not orthogonal. The genotype was regarded as a random factor. Based on this model, multiple contrast tests (Bretz et al., 2011) were conducted in order to compare the several levels of (i) variety, (ii) latitude, and (iii) experiment, respectively. Moreover, a further statistical model was established using latitude and experiment as covariates instead of the pseudo factor. On the basis of this model, an analysis of covariance (ANCOVA) was conducted (Cochran, 1957), resulting in (three) different linear regression functions with the same slope.

## Results

### Large phenotypic variation for flowering time in species of the genus *Beta*

**Figure 1:**
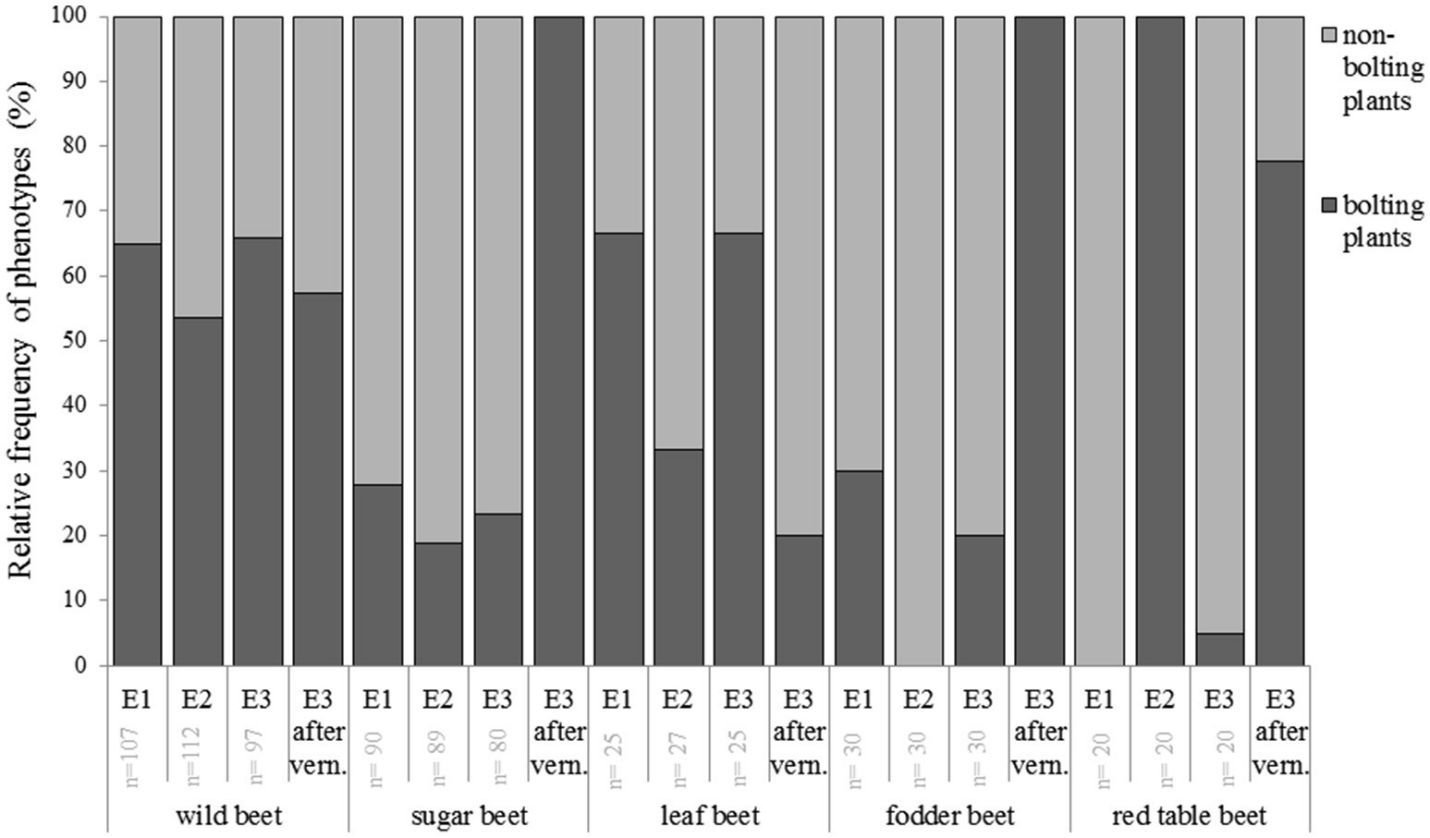
Phenological development of cultivated beet under three different environments (experiment 1–3). Bolting was determined as the beginning of shoot elongation (BBCH51; Meier et al., 1993). Plants were grown in pods in a climate chamber and kept under three different LD conditions: 22 h light, 20°C (E1), 16 h light, 20°C (E2), 22 h light, 20°C, interrupted by 12 weeks of 4°C (E3). The number of cultivars used (*n*) is indicated in light gray. In E1 and E2 “non-bolting” means that plants did not bolt until 16 weeks after sowing. In E3 “non-bolting” means that plants did not bolt until the end of the experiment (16 weeks after vernalization).

We chose 29 accessions from different geographical origin to represent the genetic diversity of the species *B. vulgaris* (Table 1). Of each accession, 10 plants were grown in a climate chamber under three different environmental conditions. The onset of bolting was assessed as beginning of elongation of the main stem (BBCH51) after Meier et al., 1993). In experiment 1 and 2, plants were held under 22 and 16 h of light, respectively. In the third experiment, the same day/night regime as in experiment 1 was applied but biennial accessions were subjected to another 12 weeks of cold treatment (4°C). We uncovered annual and biennial bolting behavior in both wild and cultivated accessions (Figure 1, Supplementary Table 4).

**Figure 2:**
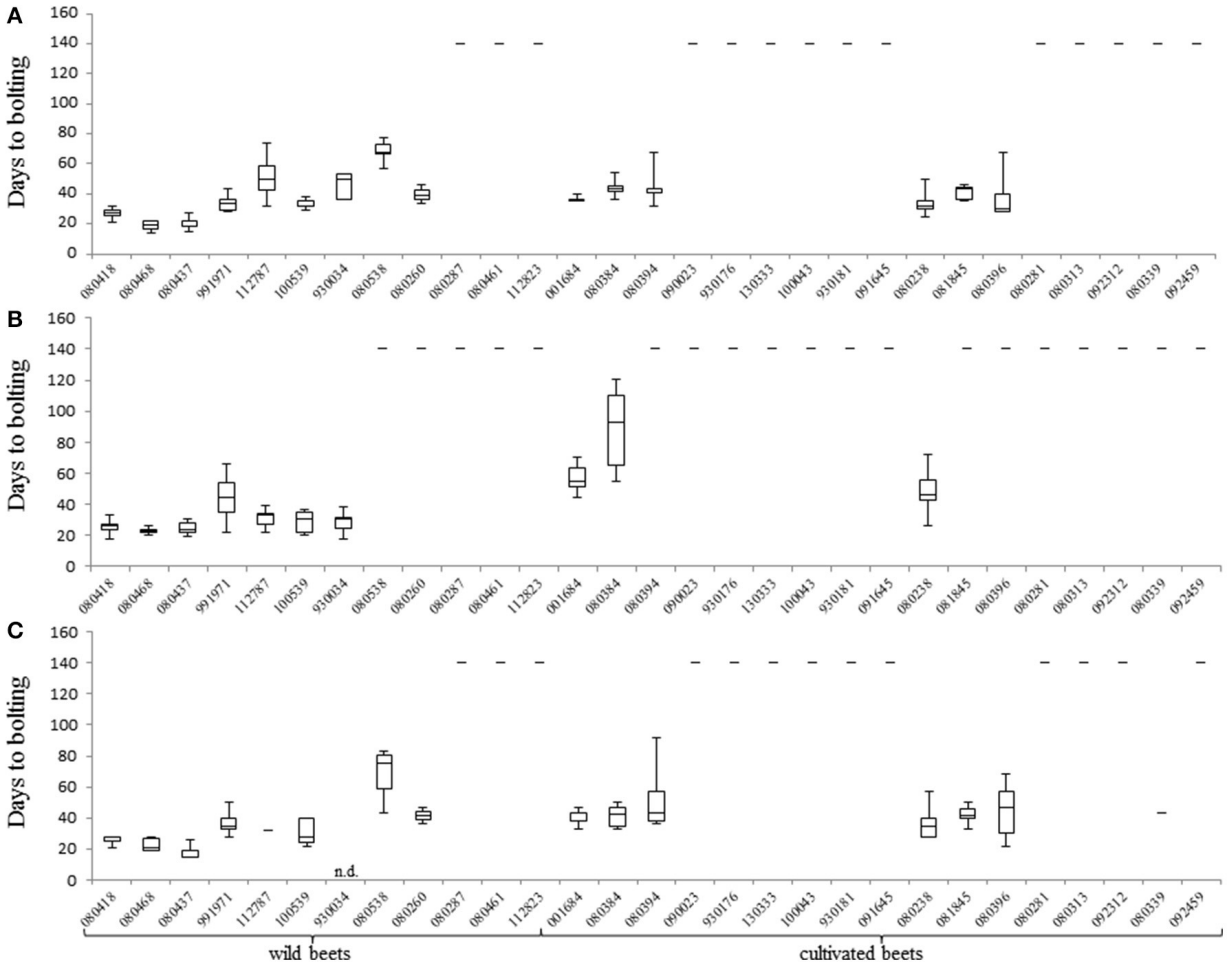
Bolting time measurements for all accessions under different environmental conditions. **(A)** Days to bolting (DTB) of plants of accessions that bolted before vernalization when held in experiment 1 (22 h light/2 h dark). **(B)** DTB of plants of accessions when held in experiment 2 (16 h light/8 h dark). **(C)** DTB of plants of accessions when held in experiment 3 (22 h light/2 h dark). Bolting was determined as DTB after sowing without vernalization. Plants were grown in pods in a climate chamber and kept under LD conditions at 20°C, 315 μmol m^−2^s^−1^ and 70% humidity. Plants are separated by wild beet accessions and cultivated beet accessions. For plants of accessions that did not bolt without vernalization the value of days to bolting was set to 140 days. Error bars represent the standard error of the mean (SEM).

There was a clear tendency for earlier bolting before vernalization under 22 h of light (experiment 1 and 3). On average, annual plants bolted 10 days earlier as when grown under 16 h of light. Six accessions were classified as annual (or segregating for annual and biennial) under 22 h of light while they behaved as biennials under 16 h of light (Figures 2A–C, Supplementary Table 4). The earliest accession, 080437 from Pakistan (31°N), bolted 19 days after sowing when grown under 22 h of light. Under 16 h of light it bolted after 27 days. The earliest accession under 16 h of light was 080468 from Egypt (27°N), which bolted 23 days after sowing. Contrary, 080538 from Great Britain (54°N) was the latest accession under 22 h of light which bolted 69 days after sowing. Interestingly, in experiment 3 only seven out of 10 plants bolted before vernalization, but the remaining three bolted after vernalization. When grown under 16 h of light, accession 080538 performed a biennial life cycle. There was considerable phenotypic variation within accessions under the same experimental conditions. The sea beet accessions 080260 (52°N), 080538 (54°N), 100539 (51°N), and 112787 (46°N) from northern latitudes segregated into annual and biennial plants (22 h of light). Similarly, the cultivated beet accessions 080384 (39°N), 080394 (32°N), and 080396 (39°N) from southern latitudes segregated for bolting under 22 h light (Supplementary Table 4).

**Figure 3:**
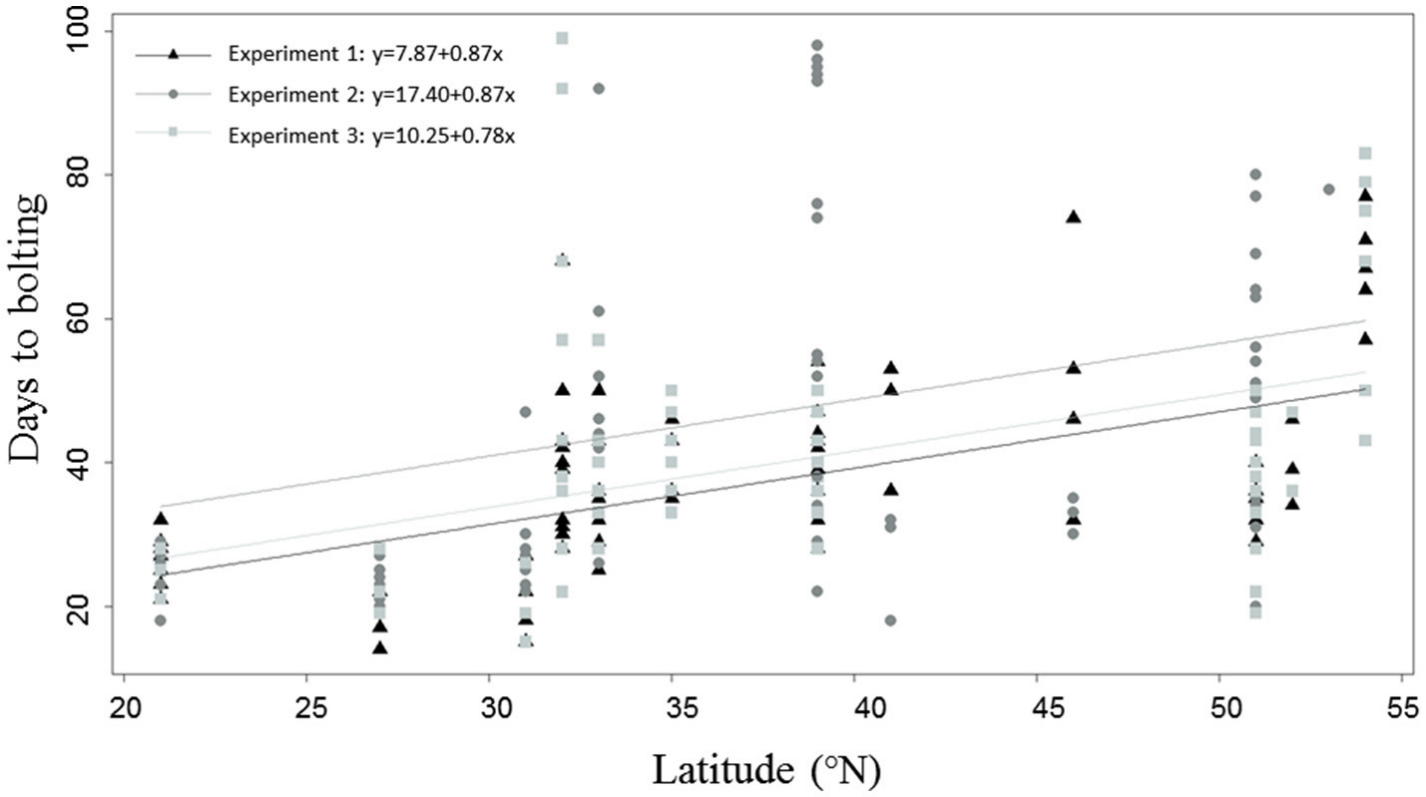
Days to bolting on a latitudinal cline (°N). Bolting plants from experiment 1 are depicted as black triangles. Bolting plants from experiment 2 are depicted as dark gray, filled circles and bolting plants from experiment 3 are depicted as light gray, filled boxes. The statistical model included experiment and latitude as covariates and an analysis of covariances (ANCOVA) was conducted, resulting in different linear regression functions for all environments with the same slope. Plants were grown in pods in a climate chamber and kept under LD conditions (either 16 h of light/8 h dark, 20°C (experiment 2) or 22 h of light/2 h dark (experiment 1 and 3), 20°C, 315 μmol m^−2^s^−1^ and 70% humidity). Non-bolting plants are not included in the analysis.

We reasoned that the phenological development of *Beta* genotypes depends on latitude of origin. To test this hypothesis, an analysis of covariances (Cochran, 1957) with data from experiment 1–3 was conducted. This analysis revealed three different linear regression functions with the same slope (Figure 3), suggesting that accessions from southern latitudes of origin flowered earlier than those from northern latitudes. As all regression functions revealed the same slope, we concluded that all environments exert a similar effect of latitude on days to bolting. Additionally, our data show that accessions from northern latitudes exhibited a tendency toward biennial bolting (Supplementary Table 4). Surprisingly, not all cultivated beets displayed a biennial behavior. The leaf beet accession 080238 from Iraq (33°N) revealed an annual life cycle under all experimental conditions (without vernalization). Similar as some sea beet accessions, it bolted earlier under 22 h light (35 days), than under 16 h light (50 days). Moreover, the sugar beet accession 080384 from Turkey (39°N) segregated for bolting and non-bolting before vernalization, while bolting plants had a strong tendency toward early flowering in all experiments. After vernalization, sugar and fodder beets bolted within 26–61 days. However, one leaf beet accession from Italy (42°N) and one table beet accession from Russia (60°N) segregated into bolting and “never bolting” after vernalization. Also, the sea beet accessions 080287, 080461, 112823, and 112787 from Ireland (53°N), Denmark (56°N), Great Britain (54°N), and France (46°N) exhibited a tendency toward “never bolting” (Supplementary Table 4).

### Haplotype variation of four major flowering time regulators

Next, we aimed to link sequence variations and phenological development. For haplotyping, the coding regions of *BTC1, BvBBX19, BvFT1*, and *BvFT2* were sequenced because they had been identified as major constituents of the bolting time regulatory pathway in beet (Pin et al., 2010, 2012; Dally et al., 2014). We sequenced pooled PCR products from single plants of an accession grown in experiment 1. If a pooled DNA sample turned out to be a mixture of different sequences or if segregation into bolting and non-bolting plants was detected, single plants were sequenced.

First, we sequenced the *BTC1* coding region (2,367 bp) for each accession and compared it to the reference sequence (Pin et al., 2012). Twenty-five out of 27 SNPs have already been described by Pin et al. (2012) whereas two additional polymorphisms (exon 8 nt2 and exon 9 nt29) turned out to be new (Table 2). Four polymorphic nucleotides are located within the sequence encoding the CCT- and the REC-domain (two in each domain). One of these (exon 3, nt351) represents a non-synonymous mutation from Asparagine to Lysine (Pin et al., 2012). In total, 1 different *BTC1* haplotypes were identified among 29 accessions. Of these, three haplotypes have been unknown so far (*BTC1*_*l*_, *BTC1*_*m*_, *BTC1*_*n*_), the remaining eight haplotypes have already been described by Pin et al. (2012) (Table 2). As expected, most of the cultivated (biennial) beet accessions carried the *btc1*_*a*_ haplotype (Supplementary Tables 5, 9), which has already been attributed as “biennial” *btc1* haplotype (*btc1*_*a*−*c*_; Pin et al., 2012).

**Table 2:** Haplotype studies with the *BTC1* gene.

**Exon**	**3**			**5**	**6**		**7**			**8**								**9**									**10**
SNP position/Haplotype	92	224	351	89	64	89	23	75	164	2	9	79	97	154	158	250	29	37	402	435	476	542	616	670	686	814	72
*btc1_*a*_*	G	**A**	**A**	**C**	G	**T**	**C**	**A**	G	**C**	**C**	**C**	**C**	G	**T**	G	G	**A**	**A**	G	**C**	G	**T**	**A**	G	**T**	**A**
*BTC1_*d*_*	G	**C**	**T**	**T**	G	G	**C**	G	**T**	**C**	**C**	**A**	**C**	G	**T**	**A**	G	**A**	G	**A**	**T**	**A**	**T**	**A**	**A**	**T**	G
*BTC1_*e*_*	G	**C**	**T**	**T**	G	G	**C**	G	**T**	**C**	**C**	**A**	**C**	G	**A**	**A**	G	**A**	G	G	**T**	**A**	**T**	**A**	G	**T**	G
*BTC1_*g*_*	G	**C**	**T**	**T**	G	G	**C**	G	**T**	**C**	**C**	**A**	**C**	G	**T**	**A**	G	**A**	G	G	**T**	**A**	**T**	**A**	G	**T**	G
*BTC1_*h*_*	G	**C**	**T**	**T**	G	G	**C**	G	**T**	**C**	**T**	**A**	**C**	G	**T**	**A**	G	**A**	G	G	**T**	**A**	**T**	**A**	G	**T**	G
*BTC1_*i*_*	G	**C**	**T**	**T**	G	G	**C**	G	G	**C**	**C**	**A**	**C**	G	**T**	**A**	G	**A**	G	G	**T**	G	**C**	G	G	**T**	G
*BTC1_*j*_*	G	**C**	**T**	**T**	**A**	G	**C**	G	**T**	**C**	**C**	**A**	**C**	G	**T**	**A**	G	**A**	G	G	**T**	G	**T**	**A**	G	**C**	G
*BTC1_*k*_*	**T**	**C**	**T**	**T**	G	G	**C**	G	**T**	**C**	**C**	**A**	**A**	G	**T**	**A**	G	**A**	G	G	**T**	G	**T**	**A**	G	**T**	G
*BTC1_*l*_*	G	**C**	**T**	**T**	G	G	**C**	G	**T**	**C**	**C**	**A**	**C**	G	**T**	**A**	**A**	**A**	G	G	**T**	**A**	**T**	**A**	G	**T**	G
*BTC1_*m*_*	G	**C**	**T**	**T**	G	G	**C**	G	G	**T**	**C**	**A**	**C**	G	**T**	**A**	G	**A**	G	G	**T**	G	**C**	**A**	G	**T**	G
*BTC1_*n*_*	G	**C**	**T**	**T**	G	G	**C**	G	**T**	**C**	**C**	**A**	**C**	G	**T**	**A**	G	**A**	G	G	**C**	G	**T**	**A**	G	**T**	G
Non-syn. SNP	^*^	^*^	^*^		^*^	^*^			^*^	^*^	^*^	^*^	^*^	^*^	^*^	^*^	^*^		^*^	^*^	^*^	^*^		^*^	^*^		

*Twenty-seven SNPs were assembled to 11 haplotypes. The coding sequence of BTC1 was sequenced from all plants of the B. vulgaris panel. The position of the SNPs is given relative to its translation start site according to the regarding exons. The different nucleotides are indicated by different colors. Asteriks represent non-synonymous SNPs*.

Second, we sequenced the coding region of the *BvBBX19* gene (588bp). Sequence variation was much lower as observed for *BTC1*. We identified one non-synonymous and three synonymous polymorphisms. As expected, none of the accession carried the EMS mutations which had been published by Dally et al. (2014). Interestingly, only one synonymous SNP was located within the region coding for the B-Box-domains. Taken together, all polymorphisms gave rise to seven haplotypes (*BvBBX19*_*a*−*g*_) across all accessions analyzed in this study (Table 3). The non-synonymous SNP was present only in two haplotypes (*BvBBX19*_*f,g*_). These haplotypes occurred in all leaf beet- and several sea beet accessions, but only in one fodder beet- and one sugar beet accession (Supplementary Table 6). Intriguingly, all accessions with the *BvBBX19*_*f*_or *BvBBX19*_*g*_haplotypes originate from southern latitudes (21–42°N).

**Table 3:** Haplotype variation within the *BvBBX19* gene.

**Exon**	**2**	**4**		
SNP position/Haplotype	69	45	59	231
*BvBBX19_*a*_*	**A**	G	**C**	**T**
*BvBBX19_*b*_*	**A**	G	**C**	**C**
*BvBBX19_*c*_*	G	G	**C**	**T**
*BvBBX19_*d*_*	G	**A**	**C**	**C**
*BvBBX19_*e*_*	G	**A**	**C**	**T**
*BvBBX19_*f*_*	G	**A**	**T**	**C**
*BvBBX19_*g*_*	G	**A**	**T**	**T**
Non-syn. SNP			^*^	

*Four SNPs were assembled to 7 haplotypes. The coding sequence of BvBBX19 was sequenced from all plants of the B. vulgaris panel. The position of the SNPs is given relative to its translation start site according to the regarding exons. The different nucleotides are indicated by different shading. Asteriks represent non-synonymous SNPs*.

Third, we analyzed the coding region of the floral repressor *BvFT1* (540 bp). In total, we found one synonymous as well as four non-synonymous SNPs. Additionally, an insertion of 3 bp in exon 1 was identified which occurred only in two accessions. These polymorphisms could be assembled to eight haplotypes (*BvFT1*_*a*−*h*_; Table 4). Four of the five polymorphisms are located outside the PEBP -domain region. Only two accessions house a polymorphism within the PEBP coding region (exon 4 nt57, haplotype *BvFT1*_*c*_; Supplementary Table 7), indicating a high conservation of this domain.

**Table 4:** Haplotype variationç within the *BvFT1* gene.

**Exon**	**1**				**4**	**ATT insertion in exon 1 btw. n111 and 112**
SNP position/Haplotype	11	20	70	72	57	
*BvFT1_*a*_*	G	**C**	**C**	**C**	**C**	
*BvFT1_*b*_*	G	**C**	G	**C**	**C**	
*BvFT1_*c*_*	G	**C**	G	**C**	**T**	
*BvFT1_*d*_*	G	**T**	G	**C**	**C**	
*BvFT1_*e*_*	G	**T**	**C**	**C**	**C**	
*BvFT1_*f*_*	**T**	**T**	**C**	**T**	**C**	X
*BvFT1_*g*_*	G	**T**	**C**	**T**	**C**	
*BvFT1_*h*_*	G	**C**	**C**	**T**	**C**	
Non-syn. SNP	^*^	^*^	^*^		^*^	

*Five SNPs were assembled to 8 haplotypes. The coding sequence of BvFT1 was sequenced from all plants of the B. vulgaris panel. The position of the SNPs is given relative to its translation start site according to the regarding exons. The different nucleotides are indicated by different colors. Asteriks represent non-synonymous SNPs*.

Fourth, the *BvFT2* gene was studied and two non-synonymous polymorphisms giving rise to four haplotypes (*BvFT2*_*a*−*d*_; Table 5) were identified. One SNP is located within the PEBP-domain region (exon 4 nt39). Remarkably, those haplotypes with the PEBP domain mutation (*BvFT2*_*b*_ and *BvFT2*_*c*_) were only present in sea beet accessions from northern latitudes (39–56°N). In contrast, *BvFT2*_*a*_ and *BvFT2*_*d*_ are highly abundant in cultivated *Beta* accessions (Supplementary Table 8).

**Table 5:** Haplotype variation within the *BvFT2* gene.

**Exon**	**1**	**4**
SNP position/Haplotype	82	39
*BvFT2_*a*_*	**A**	G
*BvFT2_*b*_*	**A**	**A**
*BvFT2_*c*_*	**C**	**A**
*BvFT2_*d*_*	**C**	G
Non-syn. SNP	^*^	^*^

*Two SNPs were assembled to 4 haplotypes. The coding sequence of BvFT2 was sequenced from all plants of the B. vulgaris panel. The position of the SNPs is given relative to its translation start site according to the regarding exons. The different nucleotides are indicated by different colors. Asteriks represent non-synonymous SNPs*.

### Relation between haplotype variation and life cycle regime

We anticipated a link between haplotype variation and life cycle regime which in turn depends on the geographical origin of an accession. First, we looked for a reciprocal relation between haplotypes and phenological development under long day conditions (experiment 1; Supplementary Table 9). As a general rule, cultivated beets which mainly exhibited a biennial life cycle displayed low genetic variation. In the majority, they carried similar combinations of their *BTC1, BvBBX19, BvFT1*, and *BvFT2* haplotypes. Most cultivated sugar beet accessions (090023, 130333, 091645, 100043, and 930176) revealed the “biennial” *btc1*_*a*_ and *BvBBX19*_*a*_ haplotypes, respectively. Moreover, seven out of nine sugar beet accessions, as well as both red table beet accessions displayed either the *BvFT1*_*a*_ and *BvFT2*_*a*_ or the *BvFT1*_*a*_ and *BvFT2*_*d*_ haplotype combination. Sea beets displayed a much higher heterogeneity (between and within accessions) regarding their *BvBBX19, BvFT1*, and *BvFT2* haplotypes whereas most accessions were fixed for only one *BTC1* haplotype (exceptions: 991971, 080538, 081845), despite a high sequence variation within this gene across all accessions. Noteworthy, the new *BTC1*_*m*_ haplotype only occurred in sea beet accessions from higher latitudes (21.0-39.0°N).

## Discussion

We examined 29 *Beta* accessions from different geographical origins including wild and cultivated beets for phenotypic plasticity under different photoperiodic conditions. Further, the haplotypes of four flowering time regulators, *BTC1, BvBBX19, BvFT1*, and *BvFT2* were analyzed to uncover the relationship between haplotype variation and life cycle adaptation. We found a general southward shift toward earlier flowering. Plants from northern latitudes flowered considerably later or did not flower at all, pointing at a strong coherence of life cycle and geographical origin. Besides, our data revealed a high conservation of the important protein domains (CCT-, REC-, BBX-, and PEBP) for all genes emphasizing their evolutionary relevance for life cycle adaptation in *Beta* species. Withal, haplotype analysis of *BvBBX19, BvFT1*, and *BvFT2* displayed only a few polymorphisms when compared with the high SNP frequency in *BTC1*. While most cultivated beets carried similar haplotype combinations of *BTC1, BvBBX19, BvFT1*, and *BvFT2*, sea beets displayed much higher heterogeneity. Our findings display several new haplotypes of beet's major floral regulators and connect these to different life cycle regimes.

To warrant evolutionary success of a flowering plant, the adaptation to different climates concomitant with life cycle control is of utmost importance. There are several environmental factors which impact phenotypic plasticity of a flowering plant, such as temperature and photoperiod (Andrés and Coupland, 2012). Species of the genus *Beta* have evolved different life cycles over the years allowing adaptation to a broad spectrum of latitudes. Besides annuality, beets have evolved biennial and perennial life cycles (Hautekèete et al., 2002), which makes this species an ideal species to study life cycle adaptation. However, prior to this study there was only scant knowledge about the relationship between plant phenology and geographical origin in correlation with genetic diversity of known flowering time genes. What are possible explanations for this relationship particularly with regard to vernalization requirement?

For sea beets (*B. vulgaris* spp. *maritima*) a genetically based latitudinal gradient for flowering time along Western European coasts has been shown, together with heritability for flowering time and vernalization requirement. Van Dijk et al. (1997) and Boudry et al. (2002) have demonstrated that differences in life cycle due to vernalization requirement seem to be an adaptive response to season length and spring temperatures in particular latitudes. Van Dijk et al. (1997) sampled seeds from 93 sea beet populations situated along a latitudinal cline around the French coast and in adjacent regions and examined flowering behavior and the relationship between latitude and vernalization requirement under greenhouse conditions. They found that the more southern the origin of plants is the less vernalization is required. While the frequency of flowering phenotypes without vernalization requirement is very high in coastal and inland populations around the Mediterranean, most plants from the Atlantic coast are not able to flower without vernalization. The authors suggested that flowering time in southern parts is controlled by warm temperatures and day length, whereas northern populations have a strong requirement for vernalization. The general importance of vernalization requirement was further emphasized by Van Dijk (2009) who showed that a shortened cold period leads to a complete inhibition of flowering which can be compensated by an artificial increase of photoperiod. Similar observations were obtained by Boudry et al. (2002), who investigated a smaller number of sea beet populations but a higher number of plants along a similar north-south cline of France as Van Dijk et al. (1997). The authors applied several cold regimes to plants of different age over 3 years in the glasshouse and in the field and found that plants from northern origins exhibit a greater requirement for vernalization. Intriguingly they detected that the northernmost population exhibited a lower reproductive success than other populations and thus seemed to be not well adapted. They hypothesized that these findings may underlie inbreeding depression and a lack of genetic variability in small rather isolated populations and that the evolutionary equilibrium has not been reached since the last ice age.

The general aspects of early flowering in wild population have been reviewed by Charnov and Schaffer (1973) who hypothesized that selection for earlier flowering and therewith reproduction underlies a decreased probability of survival. In case of sea beets, a high mortality pressure often occurs especially for inland populations in disturbed environments along roadsides or in sunflower fields, where they are under heavy selective pressure (Boudry et al., 1993). These so-called weed beets are a result of accidental cross-fertilization between ruderal wild beets and crop lineages, mainly found close to seed production fields. The selection for early flowering and a short life history based on high mortality pressure has been suggested for weed beet populations in sugar beet production areas (Van Dijk and Desplanque, 1999).

Even though several studies covered a higher number of populations and plants compared to our study, their observations mainly focus on sea beets from the Mediterranean and the Atlantic coast of France. Our *Beta* panel, by contrast, displays a broader range of genotypes from different geographical origin and it includes several cultivated beets. We detected a southward shift toward earlier flowering and a complete absence of vernalization requirement of *Beta* accessions from southern latitudes which is in line with previous studies from Boudry et al. (2002) and Van Dijk et al. (1997). Our findings show that *Beta* accessions from northern hemispheres flowered later or did not flower at all, hinting at a major effect of vernalization requirement. By contrast, accessions from southern latitudes flowered earlier, especially wild beets. This result is in line with a general observation that in southern latitudes flowering is mediated mainly by spring temperatures, while in northern latitudes winter chilling is a limiting factor (Tooke and Battey, 2010). In our study, three cultivated accessions from southern latitudes (080384, 39°N; 080394, 32°N; and 080396, 39°N) segregated into annual and biennial plants which can be explained by cross-fertilization with sea beets that suffered high mortality pressure as demonstrated by Boudry et al. (1993) and Van Dijk and Desplanque (1999). The very early flowering phenotypes we observed for some accessions from southern latitudes could be the result of southern climate conditions or environmental instability as discussed by Hautekèete et al. (2009).

How can the phenological development of *Beta* genotypes be explained by genetic variation? In a changing climate, early flowering will be selected for in long day plants (Van Dijk and Hautekèete, 2007). The direct effect of climate change on phenology in sea beets was recently demonstrated by Van Dijk and Hautekèete (2014). They sampled seeds from 73 sea beet populations on Mediterranean and European Atlantic coasts in 2 different years (1989 and 2009) and grew the plants under greenhouse conditions. As a result of natural selection within 20 years, the southern populations shifted toward later flowering, whereas the northern populations flowered earlier. The authors conclude that their findings are based on genetic changes in sensitivity to environmental cues, such as increased temperature over the years. Thus, evidence for genetic change imparting flowering phenology has been given, but the genetic reasons remained in the dark. Today we know that vernalization requirement is a key component of flowering time regulation.

In *A. thaliana*, the MADS-box gene *Flowering Locus C* (*FLC*) plays a central role in regulating vernalization response (Michaels and Amasino, 1999; Sheldon et al., 2000). In *B. vulgaris*, a vernalization-responsive *FLC* homolog, *FLC-LIKE 1* (*BvFL1*) has been identified and a conserved function as a floral repressor was suggested after genetic complementation in Arabidopsis (Reeves et al., 2007). Hébrard et al. (2013) detected RNA methylation of the *BvFL1* mRNA after vernalization which seemed to indicate its role in vernalization response. However, a recent study clearly demonstrated that RNAi-mediated down-regulation of *BvFL1* did not reveal any major effect on bolting without or after vernalization. Moreover, over-expression of *BvFL1* only led to a 1 week delay in bolting after vernalization, suggesting that *BvFL1* is not a major regulator of vernalization response in beet (Vogt et al., 2014).

Evidently, vernalization requirement is under control of the bolting locus *B* (Abegg, 1936; Van Dijk et al., 1997; Boudry et al., 2002; Van Dijk, 2009; Pin et al., 2012). Pin et al. (2012) had cloned the *BTC1* bolting gene from the *B* locus and demonstrated for the first time that natural allelic variation of a single gene impacts life cycle variation of beet. The authors suggested that life cycle adaptation results from haplotype diversity of *BTC1* which alters the plant's response to long day conditions. Interestingly, only one of the non-synonymous SNPs (exon 3, nt351) was located within the sequence encoding for the CCT-domain. This polymorphism was only present in cultivated beets which carry the *btc1*_*a*_ haplotype, indicating a potential target during domestication. In our study eleven *BTC1* haplotypes from which three were unknown to date were identified, suggesting a high genetic diversity of the chosen material. Our findings are in line with those of Pin et al. (2012) in a manner that “annual” *BTC1* haplotypes primarily occurred in sea beets from southern regions, whereas “biennial” *btc1* haplotypes were mainly found in cultivated beets from northern regions. Interestingly, we identified two sea beet accessions from northern latitudes (080287 from Ireland and 080461 from Denmark) which showed a biennial life cycle under all experimental conditions although these genotypes carried an “annual” *BTC1* haplotype.

Apart from *BTC1*, three more genes (*BvBBX19, BvFT1*, and *BvFT2*) are major flowering time regulators associated with life cycle adaptation in beet (Pin et al., 2010; Dally et al., 2014). For *BvBBX19*, it was shown that polymorphisms derived from EMS mutagenesis turned an annual to a biennial beet (Dally et al., 2014). In our study we focused on natural variation within these flowering time genes. We detected four polymorphisms within the coding region of *BvBBX19* resulting in seven haplotypes. One SNPs (exon 4, nt59) results in a non-synonymous mutation, while another one (exon 2, nt69) causes a synonymous mutation within the B-Box coding domain. The haplotypes *BvBBX19*_*f*−*g*_, harboring the non-synonymous mutation, were found exclusively in accessions from southern latitudes. Interestingly, these haplotypes appear in both, wild and cultivated beets and, with one exception, all of these accessions segregated for bolting when grow under 22 h of light. The remaining two SNPs resulted in synonymous mutations which are located outside the B-Box coding domain.

Recently, *BvFT2* was proposed as a candidate gene for seasonal bolting time at the *SBT-4* locus (Tränkner et al., 2017). We identified two and five polymorphisms within *BvFT2* and *BvFT1*, respectively. Interestingly, for each gene we found one non-synonymous SNP within the PEBP-domain coding sequence. *BvFT2* haplotypes which carried this SNP (*BvFT2*_*b*_ and *BvFT2*_*c*_) were only present in sea beets from northern latitudes (39–56°N) which primarily exhibited a biennial life cycle. In addition, the *BvFT1*_*f*_ haplotype, which displayed a 3bp insertion in the coding region of the PEBP domain, was only identified in the two southernmost sea beet accessions (080437 and 080418) from Pakistan and India which exhibited very early bolting phenotypes. We hypothesize that this insertion may impair the repressing function of *BvFT1* in these plants, thus enabling early flowering. Overall, *BvFT2* displayed the highest sequence conservation which underpins its importance as a floral integrator. This is in line with other studies where *FT* functional orthologs that induce flowering are highly conserved in diverse species, such as the rice *FT* ortholog *Heading date3* (*Hd3a*; Tamaki et al., 2007), *SINGLE FLOWER TRUSS* (*SFT*) from tomato (Lifschitz et al., 2006) or *CENTRORADIALIS8* (*ZCN8*) from *Zea mays* (Lazakis et al., 2011; Meng et al., 2011).

In conclusion, our findings show that geographical origin impacts life cycle adaptation of *Beta* genotypes. We found that vernalization requirement is absent in sea beet accessions from southern latitudes. A comparison of sequence variation of main flowering time genes between wild and cultivated beets exhibited a general tendency for increased sequence heterogeneity in sea beets. This can be explained by domestication and breeding which resulted in reduced genetic variation within these genes, indicative for selective sweeps. The *BvFT1*_*f*_ haplotype which was found in the two southernmost sea beet accessions is of great interest for further studies, because it may represent an example for evolutionary genetic change to enable a short life history due to high mortality pressure in disturbed areas as suggested by Van Dijk and Desplanque (1999). Moreover, the new *BvFT2* and *BvBBX19* haplotypes may serve as novel resource for beet breeding to broaden the variation for bolting resistance even after winter which is necessary to breed winter beets (Jung and Müller, 2009).

## Author contributions

NH: planned, conducted, and analyzed all experiments and drafted and wrote the manuscript; MH: helped with statistical analyses; ND: participated in designing the study and helped to draft the manuscript; CJ: participated in the design of the study and revised the manuscript. All authors read and approved the final manuscript.

### Conflict of interest statement

The authors declare that the research was conducted in the absence of any commercial or financial relationships that could be construed as a potential conflict of interest.

## Supplementary material

Supplementary Figure 1 *In silico* prediction of the coding gene structures of *BTC1, BvBBX19, BvFT1*, and *BvFT2*. Exons are depicted by cross-striped boxes. Conserved regions encoding for protein domains (REC., CCT-, BB1-, BB2-, and PEPB-domain) are indicated below the exonic structures. 3′- and 5′ UTRs are indicated by arrows above the exons. Identified non-synonymous polymorphisms are indicated by red arrows and synonymous polymorphisms are indicated by blue arrows.

Supplementary Table 1 List of primer combinations and PCR conditions used for sequence analysis.

Supplementary Table 2 List of markers including primer sequences used in this study for PCR and SANGER sequencing.

Supplementary Table 3 IUPAC code for incomplete nucleic acid specification (Johnson, 2010).

Supplementary Table 4 Phenotypic data from three different experiments. Plants were classified as annual (bolting within 16 weeks after sowing) or biennial (bolting only after vernalization). Plants that did not bolt 16 weeks after vernalization were classified as never bolting. Plants were grown and phenotyped in a climate chamber and kept under LD conditions with a light intensity of 900 μE.

Supplementary Table 5 *BTC1* haplotypes of all plants from the *Beta* panel. The coding region was sequenced. The position of the SNPs is given relative to the translation start site according to the regarding exons. For each accession, PCR products of 10 plants were pooled and sequenced for haplotype analysis. In case of sequence heterogeneity, all single plants were sequenced and haplotypes were assembled which could result in more than one haplotypes per accession. The nomenclature of polymorphisms was given according to the IUPAC code (Johnson, 2010).

Supplementary Table 6 *BvBBX19* haplotypes of all plants from the *Beta* panel. The coding region was sequenced. The position of the SNPs is given relative to the translation start site according to the regarding exons. For each accession, PCR products of 10 plants were pooled and sequenced for haplotype analysis. In case of sequence heterogeneity, all single plants were sequenced and haplotypes were assembled which could result in more than one haplotypes per accession.

Supplementary Table 7 *BvFT1* haplotypes of all plants from the *Beta* panel. The coding region was sequenced. The position of the SNPs is given relative to the translation start site according to the regarding exons. For each accession, PCR products of 10 plants were pooled and sequenced for haplotype analysis. In case of sequence heterogeneity, all single plants were sequenced and haplotypes were assembled which could result in more than one haplotypes per accession.

Supplementary Table 8 *BvFT2* haplotypes of all plants from the *Beta* panel. The coding region was sequenced. The position of the SNPs is given relative to the translation start site according to the regarding exons. For each accession, PCR products of 10 plants were pooled and sequenced for haplotype analysis. In case of sequence heterogeneity, all single plants were sequenced and haplotypes were assembled which could result in more than one haplotypes per accession.

Supplementary Table 9 Complete list of all haplotype combinations and phenotypic data. The table also comprises information on growth type and latitude of origin. Rows shaded in light gray indicate accessions which were non-bolting without vernalization under all environments. Cells shaded in dark gray indicate accessions which revealed a non-bolting phenotype under 16 h of light (experiment 2) but an annual phenotype or mixed phenotype (annual + biennial) under 22 h light (experiment 1+3) before vernalization.
