# Supplementary figures and images for: Haplotype Variation of Flowering Time Genes of Sugar Beet and Its Wild Relatives and the Impact on Life Cycle Regimes
Source: Front Plant Sci. 2018 Jan 4;8:2211. doi: 10.3389/fpls.2017.02211 (PMC5758561; doi:10.3389/fpls.2017.02211)

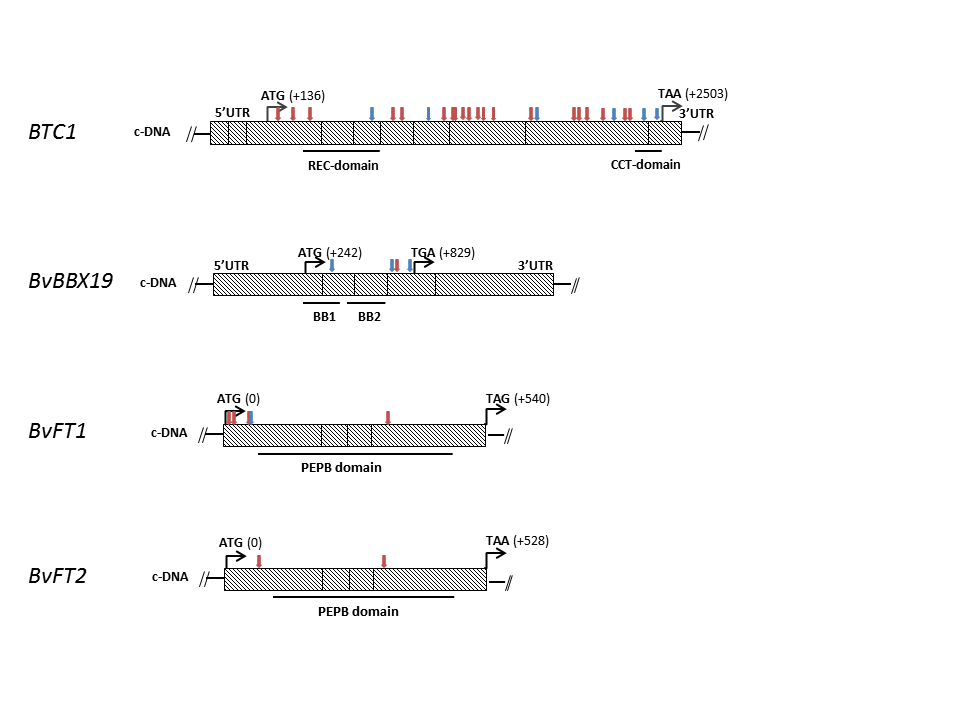

Supplement: Supplementary Figure 1 — In silico prediction of the coding gene structures of BTC1, BvBBX19, BvFT1, and BvFT2. Exons are depicted by cross-striped boxes. Conserved regions encoding for protein domains (REC., CCT-, BB1-, BB2-, and PEPB-domain) are indicated below the exonic structures. 3′- and 5′ UTRs are indicated by arrows above the exons. Identified non-synonymous polymorphisms are indicated by red arrows and synonymous polymorphisms are indicated by blue arrows. [file Image1.TIF]
