# Supplementary material for: Haplotype Variation of Flowering Time Genes of Sugar Beet and Its Wild Relatives and the Impact on Life Cycle Regimes
Source: Front Plant Sci. 2018 Jan 4;8:2211. doi: 10.3389/fpls.2017.02211 (PMC5758561; doi:10.3389/fpls.2017.02211)
Supplement: Supplementary Table 1 — List of primer combinations and PCR conditions used for sequence analysis. [file Table1.DOCX]

Supplementary Table 1. List of primer combinations and PCR conditions used for sequence analysis.

| **Target gene** | **Forward Primer** | **Reverse Primer** | **PCR conditions** |
| --- | --- | --- | --- |
| *BvBBX19* | N0162F | N0162R | 95°C, 3' + 36 x (95°C, 30"; 55°C, 30"; 72°C, 30") +72°C, 5' |
| *BvBBX19* | N0150F | N0150R | 95°C, 3' + 36 x (95°C, 30"; 55°C, 30"; 72°C, 30") +72°C, 5' |
| *BvBBX19* | NH284 | N0169R | 95°C, 3' + 36 x (95°C, 30"; 55°C, 30"; 72°C, 30") +72°C, 5'  95°C, 3' + 36 x (95°C, 30"; 55°C, 30"; 72°C, 30") +72°C, 5' |
| *BvBBX19* | N0160F | NH283 |  |
| *BTC1* | A881 | A882 | 95°C, 3' + 36 x (95°C, 30"; 57°C, 30"; 72°C, 80") +72°C, 5' |
| *BTC1* | A894 | A895 | 95°C, 3' + 36 x (95°C, 30"; 57°C, 30"; 72°C, 60") +72°C, 5' |
| *BTC1* | A884 | A886 | 95°C, 3' + 36 x (95°C, 30"; 57°C, 30"; 72°C, 60") +72°C, 5' |
| *BTC1* | A749 | A750 | 95°C, 3' + 36 x (95°C, 30"; 57°C, 30"; 72°C, 120") +72°C, 5' |
| *BvFT1* | NH340 | NH351 | 95°C, 3' + 36 x (95°C, 30"; 62°C, 30"; 72°C, 30") +72°C, 5' |
| *BvFT1* | NH338 | NH339 | 95°C, 3' + 36 x (95°C, 30"; 60°C, 30"; 72°C, 30") +72°C, 5' |
| *BvFT1* | NH334 | NH335 | 95°C, 3' + 36 x (95°C, 30"; 60°C, 30"; 72°C, 40") +72°C, 5' |
| *BvFT2* | NH362 | NH356 | 95°C, 3' + 36 x (95°C, 30"; 60°C, 30"; 72°C, 30") +72°C, 5' |
| *BvFT2* | FT2h | FT2e | 95°C, 3' + 36 x (95°C, 30"; 60°C, 30"; 72°C, 30") +72°C, 5' |
| *BvFT2* | NH358 | NH357 | 95°C, 3' + 36 x (95°C, 30"; 60°C, 30"; 72°C, 30") +72°C, 5' |
