# Supplementary material for: Haplotype Variation of Flowering Time Genes of Sugar Beet and Its Wild Relatives and the Impact on Life Cycle Regimes
Source: Front Plant Sci. 2018 Jan 4;8:2211. doi: 10.3389/fpls.2017.02211 (PMC5758561; doi:10.3389/fpls.2017.02211)
Supplement: Supplementary Table 2 — List of markers including primer sequences used in this study for PCR and SANGER sequencing. [file Table2.DOCX]

| Supplementary Table 2. List of markers including primer sequences used in this study for PCR and SANGER sequencing.   \| Target gene \| Primer name \| 5’-3’ sequence forward \| Primer  name \| 3’-5’ sequence reverse \| \| --- \| --- \| --- \| --- \| --- \| |  |
| --- | --- | --- | --- | --- | --- | --- |
| \| *BvBBX19* \| N0162F \| CAACGACGACTCAAAGAGACAG \| N0162R \| CTCAAATTTCGGGCATAGTAATG \| \| --- \| --- \| --- \| --- \| --- \| \| NH284 \| CTTCGCCCATATTGGAGATGTATC \| N0169R \| GTAAATGGAGATTTAAGAAGGTCC \| \| N0150F \| ATGCATCTTATACTATGGGACCAC \| N0150R \| GGAACAATGCTTGCCGAATCAC \| \| N0160F \| GATCTCAATGCTAGACCCCAAAG \| NH283 \| CTTCGCCCATATTGGAGATGTATC \| \| *BTC1* \| A881 \| CATACCGAAGGCGTATTCTC \| A882 \| GTGACTCATTATCTTGGACAG \| \| A894 \| ACAATGAGACCTTAGTATCCG \| A895 \| GAGCATCAAAATGGGACTGC \| \| A884 \| GTGCCAAATGGCATAGAAGC \| A886 \| CCCTAACTATCAATAGCGAGC \| \| A749 \| CAACCAGGAAAATGATGGTCG \| A750 \| CAGAATCACTGAGGTTTGACAG \| \| *BvFT1* \| NH340 \| CCATCTATACTTGTCGATGACCCTT \| NH351 \| ATTGAAATGGAGAGGTGGAATTGG \| \| NH338 \| GGAACACATCAAAAGGTGAGAAATC \| NH339 \| CTTCCATGCCCCACTACATCTTG \| \| NH334 \| GTCTCTGGAAGTTTCTTTATCGATTGG \| NH335 \| TTAGATAGTGAAGTATTCCTTAGCTTTCTAGC \| \| *BvFT2* \| NH362 \| CGATCATGCCTAGAGCACCAAGA \| NH356 \| ATCACTAAAATTATCTACTCACCAG \| \| FT2h \| CTAGTTGTCTCCCATGTTGCTAC \| FT2e \| AGCAACAATGCTCTTCAATGGCC \| \| NH358 \| GTGTAGTAAATTTACAACCTTCTTC \| NH357 \| GTGTGTAGGCCAAGAAGTTGTCTG \| | |
