# Supplementary material for: Haplotype Variation of Flowering Time Genes of Sugar Beet and Its Wild Relatives and the Impact on Life Cycle Regimes
Source: Front Plant Sci. 2018 Jan 4;8:2211. doi: 10.3389/fpls.2017.02211 (PMC5758561; doi:10.3389/fpls.2017.02211)
Supplement: Supplementary Table 3 — IUPAC code for incomplete nucleic acid specification (Johnson, 2010). [file Table3.DOCX]

Supplementary Table 3. IUPAC code for incomplete nucleic acid specification ([Johnson 2010](#_ENREF_1)).

| **Symbol** | **Mnemonic** | **Translation** |
| --- | --- | --- |
| **A** |  | A (adenine) |
| **C** |  | C (cytosine) |
| **G** |  | G (guanine) |
| **T** |  | T (thymine) |
| **U** |  | U (uracil) |
| **R** | puRine | A or G (purines) |
| **Y** | pYrimidine | C or T/U (pyrimidines) |
| **M** | aMino group | A or C |
| **K** | Keto group | G or T/U |
| **S** | Strong interaction | C or G |
| **W** | Weak interaction | A or T/U |
| **H** | not G | A, C or T/U |
| **B** | not A | C, G or T/U |
| **V** | not T/U | A, C or G |
| **D** | not C | A, G or T/U |
| **N** | aNy | A, C, G or T/U |
