# Supplementary material for: Haplotype Variation of Flowering Time Genes of Sugar Beet and Its Wild Relatives and the Impact on Life Cycle Regimes
Source: Front Plant Sci. 2018 Jan 4;8:2211. doi: 10.3389/fpls.2017.02211 (PMC5758561; doi:10.3389/fpls.2017.02211)
Supplement: Supplementary Table 4 — Phenotypic data from three different experiments. Plants were classified as annual (bolting within 16 weeks after sowing) or biennial (bolting only after vernalization). Plants that did not bolt 16 weeks after vernalization were classified as never bolting. Plants were grown and phenotyped in a climate chamber and kept under LD conditions with a light intensity of 900 μE. [file Table4.DOCX]

Supplementary Table 4. Phenotypic data from three different experiments. Plants were classified as annual (bolting within 16 weeks after sowing) or biennial (bolting only after vernalization). Plants that did not bolt 16 weeks after vernalization were classified as never bolting. Plants were grown and phenotyped in a climate chamber and kept under LD conditions with a light intensity of 900µE.

|  |  |  | Exp. 1 (22h light) | | Exp. 2 (16h light) | | Exp. 3 (22h light + vern.) | | | |
| --- | --- | --- | --- | --- | --- | --- | --- | --- | --- | --- |
| Variety | Seed code | Expected bolting behavior | Bolting without vern. | Non-bolting without vern. | Bolting without vern. | Non-bolting without vern. | Bolting without vern. | Non-bolting without vern. | Bolting after vern. | Never bolting after vern. |
|  | 080287 | biennial | - | 10 | - | 10 | - | 10 | 4 | 6 |
|  | 080461 | biennial | - | 10 | - | 10 | - | 10 | 3 | 7 |
|  | 080260 | biennial | 3 | 7 | - | 10 | 2 | 8 | 8 | - |
|  | 080538* | biennial | 8 | - | - | 10 | 7 | 3 | 3 | - |
|  | 080468 | annual | 10 | - | 10 | - | 10 | - | - | - |
| wild beet | 080437 | annual | 10 | - | 10 | - | 10 | - | - | - |
|  | 080418 | annual | 10 | - | 10 | - | 10 | - | - | - |
|  | 991971 | annual | 10 | - | 10 | - | 10 | - | - | - |
|  | 930034* | annual | 5 | - | 3 | - | - | - | - | - |
|  | 100539 | annual | 10 | - | 10 | - | 9 | 1 | 1 | - |
|  | 112787* | perennial | 4 | 4 | 3 | 6 | - | 1 | - | 1 |
|  | 112823* | perennial | - | 6 | 1 | 9 | - | 6 | 1 | 5 |
|  | 001684 | annual | 10 | - | 10 | - | 10 | - | - | - |
|  | 090023* | biennial | - | 10 | - | 10 | - | 5 | 5 | - |
|  | 930176* | biennial | - | 10 | - | 9 | - | 10 | 10 | - |
| sugar beet | 130333 | biennial | - | 10 | - | 10 | - | 7 | 7 | - |
|  | 100043* | biennial | - | 10 | - | 10 | - | 8 | 8 | - |
|  | 080394 | biennial | 7 | 3 | - | 10 | 5 | 5 | 5 | - |
|  | 930181 | biennial | - | 10 | - | 10 | - | 10 | 10 | - |
|  | 080384 | biennial | 8 | 2 | 7 | 3 | 6 | 4 | 4 | - |
|  | 091645 | biennial | - | 10 | - | 10 | - | 10 | 10 | - |
|  | 080313 | biennial | - | 10 | - | 10 | - | 10 | 10 | - |
| fodder beet | 080281 | biennial | - | 10 | - | 10 | - | 10 | 10 | - |
|  | 080396 | biennial | 9 | 1 | - | 10 | 6 | 4 | 4 | - |
| red | 092312 | biennial | - | 10 | - | 10 | 1 | 9 | 5 | 4 |
| table beet | 080339 | biennial | - | 10 | - | 10 | - | 10 | 10 |  |
|  | 080238 | biennial | 10 | - | 10 | - | 10 | - | - | - |
| leaf beet | 081845* | biennial | 7 | - | - | 10 | 10 | - | - | - |
|  | 092459* | biennial | - | 8 | - | 7 | - | 5 | 1 | 4 |
| Total |  |  | 121 | 151 | 84 | 194 | 106 | 146 | 119 | 27 |

*partly less than 10 plants due to low germination rate; vern.= vernalization; Exp.=experiment
