# Supplementary material for: Haplotype Variation of Flowering Time Genes of Sugar Beet and Its Wild Relatives and the Impact on Life Cycle Regimes
Source: Front Plant Sci. 2018 Jan 4;8:2211. doi: 10.3389/fpls.2017.02211 (PMC5758561; doi:10.3389/fpls.2017.02211)
Supplement: Supplementary Table 5 — BTC1 haplotypes of all plants from the Beta panel. The coding region was sequenced. The position of the SNPs is given relative to the translation start site according to the regarding exons. For each accession, PCR products of 10 plants were pooled and sequenced for haplotype analysis. In case of sequence heterogeneity, all single plants were sequenced and haplotypes were assembled which could result in more than one haplotypes per accession. The nomenclature of polymorphisms was given according to the IUPAC code (Johnson, 2010). [file Table5.DOCX]

**Supplementary Table 5.** ***BTC1* haplotypes of all plants from the *Beta* panel.** The coding region was sequenced. The position of the SNPs is given relative to the translation start site according to the regarding exons. For each accession, PCR products of 10 plants were pooled and sequenced for haplotype analysis. In case of sequence heterogeneity, all single plants were sequenced and haplotypes were assembled which could result in more than one haplotypes per accession. The nomenclature of polymorphisms was given according to the IUPAC code ([Johnson, 2010](#_ENREF_1)).

| **Exon** | **3** | | | **5** | **6** | | **7** | | | **8** | | | | | | | **9** | | | | | | | | | | **10** |  |
| --- | --- | --- | --- | --- | --- | --- | --- | --- | --- | --- | --- | --- | --- | --- | --- | --- | --- | --- | --- | --- | --- | --- | --- | --- | --- | --- | --- | --- |
| Seed code | 92 | 224 | 351 | 89 | 64 | 89 | 23 | 75 | 164 | 2 | 9 | 79 | 97 | 154 | 158 | 250 | 29 | 37 | 402 | 435 | 476 | 542 | 616 | 670 | 686 | 814 | 72 | Haplotypes in 10 plants / accession |
| 090023 | **G** | **A** | **A** | **C** | **G** | **T** | **C** | **A** | **G** | **C** | **C** | **C** | **C** | **G** | **T** | **G** | **G** | **A** | **A** | **G** | **C** | **G** | **T** | **A** | **G** | **T** | **A** | *btc1_a_* |
| 930176 | **G** | **A** | **A** | **C** | **G** | **T** | **C** | **A** | **G** | **C** | **C** | **C** | **C** | **G** | **T** | **G** | **G** | **A** | **A** | **G** | **C** | **G** | **T** | **A** | **G** | **T** | **A** | *btc1_a_* |
| 130333 | **G** | **A** | **A** | **C** | **G** | **T** | **C** | **A** | **G** | **C** | **C** | **C** | **C** | **G** | **T** | **G** | **G** | **A** | **A** | **G** | **C** | **G** | **T** | **A** | **G** | **T** | **A** | *btc1_a_* |
| 091645 | **G** | **A** | **A** | **C** | **G** | **T** | **C** | **A** | **G** | **C** | **C** | **C** | **C** | **G** | **T** | **G** | **G** | **A** | **A** | **G** | **C** | **G** | **T** | **A** | **G** | **T** | **A** | *btc1_a_* |
| 100043 | **G** | **A** | **A** | **C** | **G** | **T** | **C** | **A** | **G** | **C** | **C** | **C** | **C** | **G** | **T** | **G** | **G** | **A** | **A** | **G** | **C** | **G** | **T** | **A** | **G** | **T** | **A** | *btc1_a_* |
| 092312 | **G** | **A** | **A** | **C** | **G** | **T** | **C** | **A** | **G** | **C** | **C** | **C** | **C** | **G** | **T** | **G** | **G** | **A** | **A** | **G** | **C** | **G** | **T** | **A** | **G** | **T** | **A** | *btc1_a_* |
| 080339 | **G** | **A** | **A** | **C** | **G** | **T** | **C** | **A** | **G** | **C** | **C** | **C** | **C** | **G** | **T** | **G** | **G** | **A** | **A** | **G** | **C** | **G** | **T** | **A** | **G** | **T** | **A** | *btc1_a_* |
| 080281 | **G** | **A** | **A** | **C** | **G** | **T** | **C** | **A** | **G** | **C** | **C** | **C** | **C** | **G** | **T** | **G** | **G** | **A** | **A** | **G** | **C** | **G** | **T** | **A** | **G** | **T** | **A** | *btc1_a_* |
| 001684 | **G** | **C** | **T** | **T** | **G** | **G** | **C** | **G** | **T** | **C** | **C** | **A** | **C** | **G** | **T** | **A** | **G** | **A** | **G** | **A** | **T** | **A** | **T** | **A** | **A** | **T** | **G** | *BTC1_d_* |
| 100539 | **G** | **C** | **T** | **T** | **G** | **G** | **C** | **G** | **T** | **C** | **C** | **A** | **C** | **G** | **A** | **A** | **G** | **A** | **G** | **G** | **T** | **A** | **T** | **A** | **G** | **T** | **G** | *BTC1_e_* |
| 930034 | **G** | **C** | **T** | **T** | **G** | **G** | **C** | **G** | **T** | **C** | **C** | **A** | **C** | **G** | **A** | **A** | **G** | **A** | **G** | **G** | **T** | **A** | **T** | **A** | **G** | **T** | **G** | *BTC1_e_* |
| 080384 | **G** | **C** | **T** | **T** | **G** | **G** | **C** | **G** | **T** | **C** | **C** | **A** | **C** | **G** | **A** | **A** | **G** | **A** | **G** | **G** | **T** | **A** | **T** | **A** | **G** | **T** | **G** | *BTC1_e_* |
| 092459 | **G** | **C** | **T** | **T** | **G** | **G** | **C** | **G** | **T** | **C** | **C** | **A** | **C** | **G** | **A** | **A** | **G** | **A** | **G** | **G** | **T** | **A** | **T** | **A** | **G** | **T** | **G** | *BTC1_e_* |
| 080238 | **G** | **C** | **T** | **T** | **G** | **G** | **C** | **G** | **T** | **C** | **C** | **A** | **C** | **G** | **T** | **A** | **G** | **A** | **G** | **G** | **T** | **A** | **T** | **A** | **G** | **T** | **G** | *BTC1_g_* |
| 080396 | **G** | **C** | **T** | **T** | **G** | **G** | **C** | **G** | **T** | **C** | **T** | **A** | **C** | **G** | **T** | **A** | **G** | **A** | **G** | **G** | **T** | **A** | **T** | **A** | **G** | **T** | **G** | *BTC1_h_* |
| 080461 | **G** | **C** | **T** | **T** | **A** | **G** | **C** | **G** | **T** | **C** | **C** | **A** | **C** | **T** | **T** | **A** | **G** | **A** | **G** | **G** | **T** | **G** | **T** | **A** | **G** | **C** | **G** | *BTC1_j_* |
| 080287 | **G** | **C** | **T** | **T** | **A** | **G** | **C** | **G** | **T** | **C** | **C** | **A** | **C** | **T** | **T** | **A** | **G** | **A** | **G** | **G** | **T** | **G** | **T** | **A** | **G** | **C** | **G** | *BTC1_j_* |
| 112787 | **T** | **C** | **W** | **T** | **G** | **G** | **C** | **G** | **T** | **C** | **C** | **A** | **A** | **G** | **T** | **A** | **G** | **A** | **G** | **G** | **T** | **G** | **T** | **A** | **G** | **T** | **G** | *BTC1_k_* |
| 112823 | **T** | **C** | **W** | **T** | **G** | **G** | **C** | **G** | **T** | **C** | **C** | **A** | **A** | **G** | **T** | **A** | **G** | **A** | **G** | **G** | **T** | **G** | **T** | **A** | **G** | **T** | **G** | *BTC1_k_* |
| 080260 | **T** | **C** | **W** | **T** | **G** | **G** | **C** | **G** | **T** | **C** | **C** | **A** | **M** | **K** | **T** | **A** | **G** | **A** | **G** | **G** | **T** | **G** | **T** | **A** | **G** | **T** | **G** | *BTC1_k_* |
| 930181 | **G** | **C** | **T** | **T** | **G** | **G** | **C** | **G** | **T** | **C** | **C** | **A** | **C** | **G** | **T** | **A** | **A** | **A** | **G** | **G** | **T** | **A** | **T** | **A** | **G** | **T** | **G** | *BTC1_l_* |
| 080313 | **G** | **C** | **T** | **T** | **G** | **G** | **C** | **G** | **T** | **C** | **C** | **A** | **C** | **G** | **T** | **A** | **A** | **A** | **G** | **G** | **T** | **A** | **T** | **A** | **G** | **T** | **G** | *BTC1_l_* |
| 080468 | **G** | **C** | **T** | **T** | **G** | **G** | **C** | **G** | **G** | **T** | **C** | **A** | **C** | **G** | **T** | **A** | **G** | **A** | **G** | **G** | **T** | **G** | **C** | **A** | **G** | **T** | **G** | *BTC1_m_* |
| 080437 | **G** | **C** | **T** | **T** | **G** | **G** | **C** | **G** | **G** | **T** | **C** | **A** | **C** | **G** | **T** | **A** | **G** | **A** | **G** | **G** | **T** | **G** | **C** | **A** | **G** | **T** | **G** | *BTC1_m_* |
| 080418 | **G** | **C** | **T** | **T** | **G** | **G** | **C** | **G** | **G** | **T** | **C** | **A** | **C** | **G** | **T** | **A** | **G** | **A** | **G** | **G** | **T** | **G** | **C** | **A** | **G** | **T** | **G** | *BTC1_m_* |
| 991971 | **G** | **C** | **T** | **T** | **G** | **G** | **C** | **G** | **G** | **Y** | **C** | **A** | **C** | **G** | **W** | **A** | **G** | **R** | **G** | **G** | **T** | **G** | **Y** | **A** | **G** | **T** | **G** | *BTC1_e_, BTC1_f_, BTC1_m_* |
| 080394 | **G** | **C** | **T** | **T** | **G** | **G** | **C** | **G** | **K** | **C** | **C** | **A** | **C** | **G** | **T** | **A** | **G** | **A** | **G** | **G** | **C** | **G** | **T** | **A** | **G** | **T** | **G** | *BTC1_n_* |
| 080538 | **G** | **C** | **T** | **T** | **R** | **G** | **C** | **G** | **K** | **C** | **C** | **A** | **C** | **K** | **T** | **A** | **G** | **A** | **G** | **G** | **Y** | **G** | **T** | **A** | **G** | **M** | **G** | *BTC1_j_, BTC1_n_* |
| 081845 | **G** | **C** | **T** | **T** | **G** | **G** | **C** | **G** | **K** | **C** | **C** | **A** | **C** | **G** | **T** | **A** | **G** | **A** | **G** | **G** | **Y** | **G** | **Y** | **A** | **R** | **T** | **G** | *BTC1_i_, BTC1_m,_ BTC1_n_* |
| non-syn. SNP | ***** | ***** | ***** |  | ***** | ***** | ***** |  | ***** |  | ***** | ***** | ***** | ***** | ***** | ***** | ***** |  | ***** | ***** | ***** | ***** |  | ***** | ***** |  |  |  |
