# Supplementary material for: Haplotype Variation of Flowering Time Genes of Sugar Beet and Its Wild Relatives and the Impact on Life Cycle Regimes
Source: Front Plant Sci. 2018 Jan 4;8:2211. doi: 10.3389/fpls.2017.02211 (PMC5758561; doi:10.3389/fpls.2017.02211)
Supplement: Supplementary Table 9 — Complete list of all haplotype combinations and phenotypic data. The table also comprises information on growth type and latitude of origin. Rows shaded in light gray indicate accessions which were non-bolting without vernalization under all environments. Cells shaded in dark gray indicate accessions which revealed a non-bolting phenotype under 16 h of light (experiment 2) but an annual phenotype or mixed phenotype (annual + biennial) under 22 h light (experiment 1+3) before vernalization. [file Table9.DOCX]

Supplementary Table 9. Complete list of all haplotype combinations and phenotypic data. The table also comprises information on growth type and latitude of origin. Rows shaded in light grey indicate accessions which were non-bolting without vernalization under all environments. Cells shaded in dark grey indicate accessions which revealed a non-bolting phenotype under 16 hours of light (experiment 2) but an annual phenotype or mixed phenotype (annual + biennial) under 22 hours light (experiment 1+3) before vernalization.

|  |  |  |  |  |  |  | **Experiment 1 (22h light)** | | **Experiment 2 (16h light)** | | **Experiment 3 (22h light+vernalization)** | | | |
| --- | --- | --- | --- | --- | --- | --- | --- | --- | --- | --- | --- | --- | --- | --- |
| **Type** | **Seed Code** | **Latitude of origin (°N)** | ***BTC1* haplotype** | ***BvBBX19* haplotype** | ***BvFT1* haplotype** | ***BvFT2* haplotype** | Bolting without vern. | Non-bolting without vern. | Bolting without vern. | Non-bolting without vern. | Bolting without vern. | Non-bolting without vern. | Bolting after vern. | Never bolting after vern. |
| sugar beet | 090023 | 51.0 | *btc1_a_* | *BvBBX19_a_* | *BvFT1_a_* | *BvFT2_d_* | - | 10 | - | 10 | - | 5 | 5 | - |
|  | 130333 | 51.0 | *btc1_a_* | *BvBBX19_a_* | *BvFT1_a_* | *BvFT2_d_* | - | 10 | - | 10 | - | 7 | 7 | - |
|  | 091645 | 51.0 | *btc1_a_* | *BvBBX19_a_* | *BvFT1_a_* | *BvFT2_d_* | - | 10 | - | 10 | - | 10 | 10 | - |
|  | 100043 | 51.0 | *btc1_a_* | *BvBBX19_a_* | *BvFT1_a_* | *BvFT2_a_* | - | 10 | - | 10 | - | 8 | 8 | - |
|  | 930176 | 51.0 | *btc1_a_* | *BvBBX19_a_* | *BvFT1_b_* | *BvFT2_d_* | - | 10 | - | 9 | - | 10 | 10 | - |
|  | 930181 | 45.0 | *BTC1_l_* | *BvBBX19_e_* | *BvFT1_a_* | *BvFT2_a_, BvFT2_d_* | - | 10 | - | 10 | - | 10 | 10 | - |
|  | 001684 | 51.0. | *BTC1_d_* | *BvBBX19_a_* | *BvFT1_a_* | *BvFT2_a_* | 10 | - | 10 | - | 10 | - | - | - |
|  | 080384 | 39.0 | *BTC1_e_* | *BvBBX19_e_* | *BvFT1_a_* | *BvFT2_d_* | 8 | 2 | 7 | 3 | 6 | 4 | 4 | - |
|  | 080394 | 32.0 | *BTC1_n_* | *BvBBX19_d_, BvBBX19_f_* | *BvFT1_g_, BvFT1_h_* | *BvFT2_d_* | 7 | 3 | - | 10 | 5 | 5 | 5 | - |
| red table beet | 092312 | 60.0 | *btc1_a_* | *BvBBX19_a_* | *BvFT1_a_* | *BvFT2_d_* | - | 10 | - | 10 | 1 | 9 | 5 | 4 |
|  | 080339 | 45.5 | *btc1_a_* | *BvBBX19_a_* | *BvFT1_a_, BvFT1_b_* | *BvFT2_a_, BvFT2_d_* | - | 10 | - | 10 | - | 10 | 10 |  |
| fodder beet | 080281 | 51.0 | *btc1_a_* | *BvBBX19_d_* | *BvFT1_a_, BvFT1_b_* | *BvFT2_d_* | - | 10 | - | 10 | - | 10 | 10 | - |
|  | 080313 | 39.0 | *BTC1_l_* | *BvBBX19_c_, BvBBX19_d_* | *BvFT1_a_* | *BvFT2_d_* | - | 10 | - | 10 | - | 10 | 10 | - |
|  | 080396 | 32.0 | *BTC1_h_* | *BvBBX19_f_* | *BvFT1_a_* | *BvFT2_a_* | 9 | 1 | - | 10 | 6 | 4 | 4 | - |
| leaf beet | 092459 | 42.0 | *BTC1_e_* | *BvBBX19_f_* | *BvFT1_b_, BvFT1_c_* | *BvFT2_a_* | - | 8 | - | 7 | - | 5 | 1 | 4 |
|  | 080238 | 33.0 | *BTC1_g_* | *BvBBX19_c_, BvBBX19_f_, BvBBX19_g_* | *BvFT1_h_* | *BvFT2_d_* | 10 | - | 10 | - | 10 | - | - | - |
|  | 081845 | 35.0 | *BTC1_i,_ BTC1_m,_ BTC1_n_* | *BvBBX19_f_, BvBBX19_g_* | *BvFT1_a_, BvFT1_b_* | *BvFT2_a_, BvFT2_d_* | 7 | - | - | 10 | 10 | - | - | - |
| wild beet | 080287 | 53.0 | *BTC1_j_* | *BvBBX19_d_* | *BvFT1_a_, BvFT1_e_* | *BvFT2_d_, BvFT2_c_* | - | 10 | - | 10 | - | 10 | 4 | 6 |
|  | 080461 | 56.0 | *BTC1_j_* | *BvBBX19_d_* | *BvFT1_a_, BvFT1_e_* | *BvFT2_d_, BvFT2_c_* | - | 10 | - | 10 | - | 10 | 3 | 7 |
|  | 112823 | 54.0 | *BTC1_k_* | *BvBBX19_a_* | *BvFT1_a_, BvFT1_b_* | *BvFT2_d_, BvFT2_c_* | - | 6 | 1 | 9 | - | 6 | 1 | 5 |
|  | 100539 | 51.0 | *BTC1_e_* | *BvBBX19_a_* | *BvFT1_a_* | *BvFT2_a_, BvFT2_b_* | 10 | - | 10 | - | 9 | 1 | 1 | - |
|  | 930034 | 40.0 | *BTC1_e_* | *BvBBX19_e_* | *BvFT1_a_* | *BvFT2_a_, BvFT2_b_* | 5 | - | 3 | - | - | - | - | - |
|  | 080468 | 27.0 | *BTC1_m_* | *BvBBX19_f_* | *BvFT1_c_* | *BvFT2_a_, BvFT2_d_* | 10 | - | 10 | - | 10 | - | - | - |
|  | 080437 | 31.0 | *BTC1_m_* | *BvBBX19_d_, BvBBX19_f_* | *BvFT1_f_* | *BvFT2_a_* | 10 | - | 10 | - | 10 | - | - | - |
|  | 080418 | 21.0 | *BTC1_m_* | *BvBBX19_d_, BvBBX19_f_* | *BvFT1_f_* | *BvFT2_a_* | 10 | - | 10 | - | 10 | - | - | - |
|  | 991971 | 39.0 | *BTC1_m_* | *BvBBX19_d_, BvBBX19_f_, BvBBX19_g_* | *BvFT1_a_* | *BvFT2_a_, BvFT2_b_, BvFT2_d_* | 10 | - | 10 | - | 10 | - | - | - |
|  | 080260 | 52.0 | *BTC1_k_* | *BvBBX19_a_, BvBBX19_d_* | *BvFT1_b_, BvFT1_d_* | *BvFT2_a_, BvFT2_b_, BvFT2_d_* | 3 | 7 | - | 10 | 2 | 8 | 8 | - |
|  | 112787 | 46.0 | *BTC1_k_* | *BvBBX19_a_, BvBBX19_b_* | *BvFT1_a_, BvFT1_b_, BvFT1_d_* | *BvFT2_a_, BvFT2_b_, BvFT2_c_, BvFT2_d_* | 4 | 4 | 3 | 6 | - | 1 | - | 1 |
|  | 080538 | 54.0 | *BTC1_j_, BTC1_n_* | *BvBBX19_d_* | *BvFT1_a_, BvFT1_e_* | *BvFT2_c_* | 8 | - | - | 10 | 7 | 3 | 3 | - |
